# Developing remote patient monitoring infrastructure using commercially available cloud platforms

**DOI:** 10.3389/fdgth.2024.1399461

**Published:** 2024-11-06

**Authors:** Minh Cao, Ramin Ramezani, Vivek Kumar Katakwar, Wenhao Zhang, Dheeraj Boda, Muneeb Wani, Arash Naeim

**Affiliations:** ^1^ Center for Smart Health, University of California Los Angeles, Los Angeles, CA, United States; ^2^ UCLA Health Information Technology, University of California Los Angeles, Los Angeles, CA, United States; ^3^ David Geffen UCLA School of Medicine, University of California Los Angeles, Los Angeles, CA, United States

**Keywords:** big data analytics, cloud computing, healthcare, internet of things (IoT), wireless sensor networks, remote patient monitoring, AI

## Abstract

Wearable sensor devices for continuous patient monitoring produce a large volume of data, necessitating scalable infrastructures for efficient data processing, management and security, especially concerning Patient Health Information (PHI). Adherence to the Health Insurance Portability and Accountability Act (HIPAA), a legislation that mandates developers and healthcare providers to uphold a set of standards for safeguarding patients’ health information and privacy, further complicates the development of remote patient monitoring within healthcare ecosystems. This paper presents an Internet of Things (IoT) architecture designed for the healthcare sector, utilizing commercial cloud platforms like Microsoft Azure and Amazon Web Services (AWS) to develop HIPAA-compliant health monitoring systems. By leveraging cloud functionalities such as scalability, security, and load balancing, the architecture simplifies the creation of infrastructures adhering to HIPAA standards. The study includes a cost analysis of Azure and AWS infrastructures and evaluates data processing speeds and database query latencies, offering insights into their performance for healthcare applications.

## 1 Introduction

In recent years, the proliferation of affordable smart devices, including smartphones, smartwatches, and Bluetooth-enabled sensors, has paved the way for the emergence of mobile health applications. These devices incorporate various sensors such as accelerometers, gyroscopes, proximity beacons, and ECG sensors, some of which are integrated into smartwatches or smartphones. They have the capability to capture patients’ real-time activity and pathophysiological information with high accuracy (1–4). Furthermore, they often possess predictive capabilities, enabling the anticipation of patient outcomes in advance (5–7). These devices can function as standalone wireless sensors or operate alongside other wireless sensors to create a sensor network that collects and preprocesses the data before uploading it to a cloud service. The interaction among these components with the cloud service forms the backbone of a remote patient monitoring system (RPM).

As the number of sensors in the RPM system increases, scalability becomes crucial to handle the growing data volume while adhering to healthcare security standards. However, constructing and maintaining scalable infrastructures, along with developing patient monitoring algorithms, can be overwhelming and challenging for companies or research institutes primarily focused on patient care. To address this challenge, leveraging cloud computing services from available commercial cloud providers is essential. Major cloud platforms such as Microsoft Azure and Amazon Web Services (AWS) provide the necessary infrastructure modules suitable for remote patient monitoring solutions, including IoT hub, data storage, computing, and web application hosting. To fully capitalize on the scalability offered by cloud infrastructure, it is advisable to design the RPM system using the microservice architecture. This architectural approach involves breaking down the large application into multiple smaller modular applications that can be developed independently. By adopting this approach, the system can effectively leverage the benefits of the emerging container technology, allowing software developers to deploy their applications in any environment, regardless of the underlying infrastructure. This strategy reduces the technical complexities associated with deploying, managing, and scaling cloud applications. Furthermore, it enables healthcare applications to be deployed across various cloud platforms while complying with data security standards, such as those outlined in the HIPAA guidelines.

**Figure 1:**
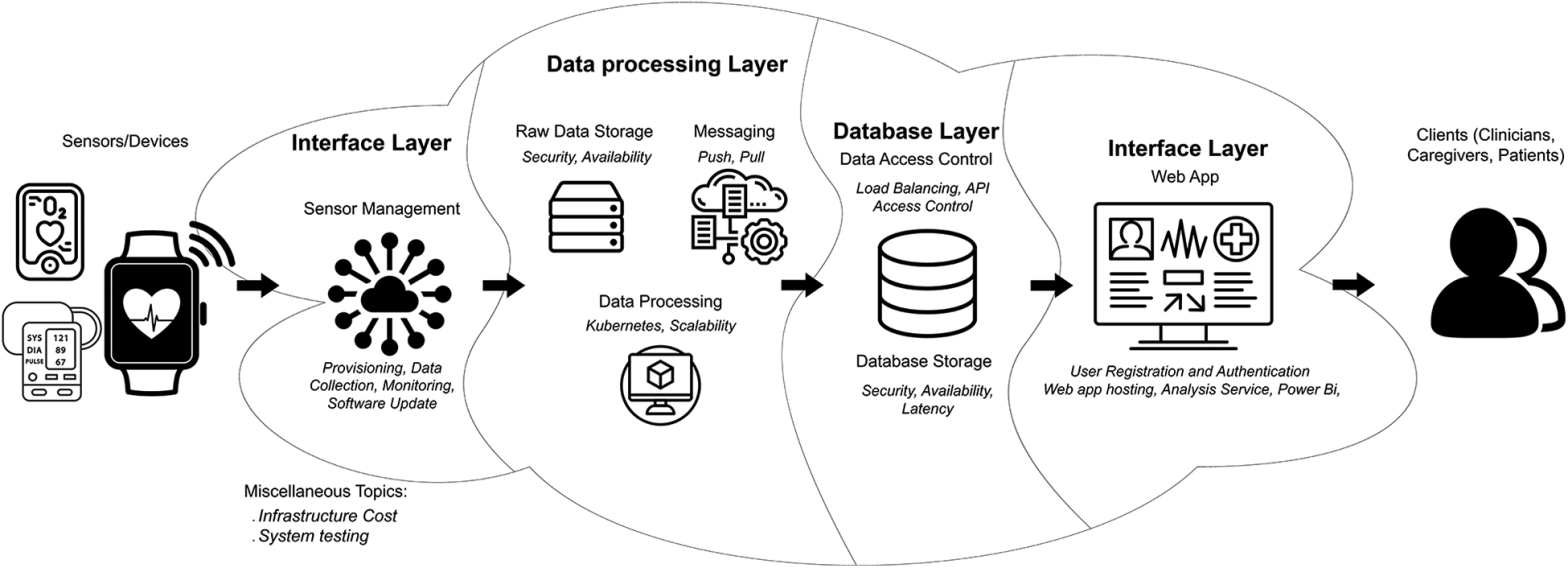
Overview of the proposed cloud architecture. The data from the sensor will be collected by the sensor hub at the interface layer to be processed at the data processing layer. Once the processing is done, the data will be stored long-term at the database layer. The Role-Based Access Controlled (RBAC) client will fetch their assigned data from the database layer. This figure provides a quick overview of the sections and subsections discussed in this paper.

This paper presents a proposal for a HIPAA-compliant RPM architecture that encompasses the collection, analysis, and visualization of patients’ data, with the ability to scale automatically as needed. The paper focuses on discussing the key aspects of such systems and outlines different approaches to leverage available services on major cloud platforms, specifically Azure and AWS, for the development and deployment of an IoT-based healthcare infrastructure. It provides recommendations on design decisions and implementation details related to security, HIPAA compliance, and the utilization of container technology within cloud platforms. Additionally, the paper explores essential criteria for selecting cloud providers, including considerations such as security, ease of deployment, and scalability. Figure 1 offers a concise summary of the primary topics addressed in the sections and subsections of this paper.

## 2 Background

### 2.1 Internet of things

The Internet of Things (IoT) refers to a system that enables the exchange of data between a network of physical sensors and a central server (8). In healthcare applications, this network of physical hardware sensors captures various types of data, including accelerometer readings, heart rate measurements, blood oxygen levels, Bluetooth proximity beacons, survey responses, and more. The collected data is typically buffered locally before being uploaded to a remote server at fixed intervals. The integration of IoT devices has gained momentum due to the widespread adoption of low-energy communication technologies like Bluetooth and Zigbee, fostering the growth of the market for affordable personal healthcare sensors. In healthcare applications, it is crucial to collect, store, and transmit sensor data securely in compliance with guidelines such as HIPAA. Streamlining these data collection processes can be achieved by leveraging prebuilt cloud modules and software development kits offered by commercially available cloud platform providers like Azure and AWS.

### 2.2 HIPAA

The Health Insurance Portability and Accountability Act of 1996 (HIPAA) is a US federal law that establishes standards for data security and privacy of medical records (9). It is imperative for any application that handles patients’ data to adhere to HIPAA guidelines. In the context of IoT systems, the following aspects of HIPAA are particularly significant (10, 11):

a) **Access control:** Both physical and electronic access to medical data should be restricted to authorized personnel or applicable patients only.
b) **Secure encryption:** Medical data must be encrypted to ensure its security while at rest and during transit.
c) **Audit report:** Every interaction with the data should be logged, including details of the party that requested the data and the specific data accessed.
d) **Backup:** The data stored remotely should be regularly backed up to ensure that it can be fully recovered in the event of disasters or failures.

Complying with these HIPAA requirements is crucial for maintaining the confidentiality, integrity, and availability of patients’ medical data within IoT systems.

### 2.3 Challenges in remote monitoring system

The proliferation of IoT devices and cloud service providers has revolutionized remote patient monitoring, reducing the need for frequent doctor visits. Patients can now upload data collected from sensors, such as smartwatches and various Bluetooth-connected devices, directly to the cloud from home for review by healthcare professionals (1–7). These devices can monitor a range of health metrics including blood pressure, glucose levels, heart rate, oxygen saturation, respiratory rate, and sleep cycles, storing unstructured data in the cloud for detailed health tracking. This system not only minimizes patient visits and lowers personnel and administrative costs but also enhances patients’ quality of life by freeing up time for other activities. However, the challenge lies in designing a scalable, secure system for data transmission and storage that can process unstructured data into a visualizable format on a dashboard. The following sections will discuss the architecture and technology used to achieve these objectives.

### 2.4 Design philosophy: microservices architecture

Traditionally, server applications have been developed using a monolithic approach, where a single application encompasses all the services and functionalities of the entire project (12). For instance, in an IoT application that aggregates data collected from multiple client devices, the monolithic architecture would be a single monolithic web server application responsible for data retrieval, processing logic, and database updates. This tightly integrated structure allows for performance optimization. However, it also introduces significant complexity, making it challenging for a single developer to handle. Modifying the system becomes burdensome as any changes necessitate extensive testing of the entire system.

An alternative architecture has emerged, known as microservices, which involves breaking down the monolithic application into more manageable services (13). Each service has its own set of Application Programming Interfaces (APIs) that enable other services to interact with it without requiring knowledge of its internal workings. This interface allows each service to function as an independent entity that can be developed, tested, and updated separately. Decomposing the monolith into smaller modules reduces technical challenges and facilitates faster iteration between updates. Additionally, since each component acts independently, scaling and reusability across platforms become highly flexible. This modular approach has been widely adopted by cloud platform providers. In the subsequent section, we propose the utilization of various modularized services provided by Azure and AWS in our system architecture (14, 15).

### 2.5 Scalability via container technology

Traditional cloud applications rely on VMs as the foundation. A VM utilizes a hypervisor, which operates on top of the host operating system (OS) and provides hardware abstraction (13). This abstraction enables users to interact with an isolated OS environment without needing knowledge of the underlying hardware. This technology forms the basis of cloud platforms, allowing cloud providers to share computing resources among multiple users while providing each user with their independent OS environment.

A more recent virtualization scheme, container-based virtualization, has emerged as an alternative. It enables the rapid deployment of cross-platform applications by creating a separate environment that runs as an application on top of the host OS. Unlike traditional VMs, container-based virtualization does not require a separate OS environment for each application. Instead, users package the application along with its dependencies into an image file, which can then be executed as an application on various host OS environments supported by container technology. Containerized applications offer better performance compared to VMs since they run on the same OS as the host and consume resources as needed, avoiding the fixed allocation of resources and the use of isolated Guest OS (14, 15). Consequently, we recommend leveraging container technology to develop efficient cross-platform IoT applications.

### 2.6 Kubernetes services basic concept

To automate the deployment, management, and scaling of cloud applications using container technology, we utilize Kubernetes. Kubernetes is an open-source container-orchestration system that automates software deployment, scaling, and management (16). It simplifies the configuration of computing resources, requiring only a few parameters like the number of CPU cores, RAM, and the maximum allowed number of running container instances. After developers set these parameters, Kubernetes takes charge of load balancing, routing, ingress control (restricting container access to authorized applications), and auto-scaling. Supported by major cloud platforms such as Azure, AWS, and Google, Kubernetes serves as an ideal cross-platform resource management system.

**Figure 2:**
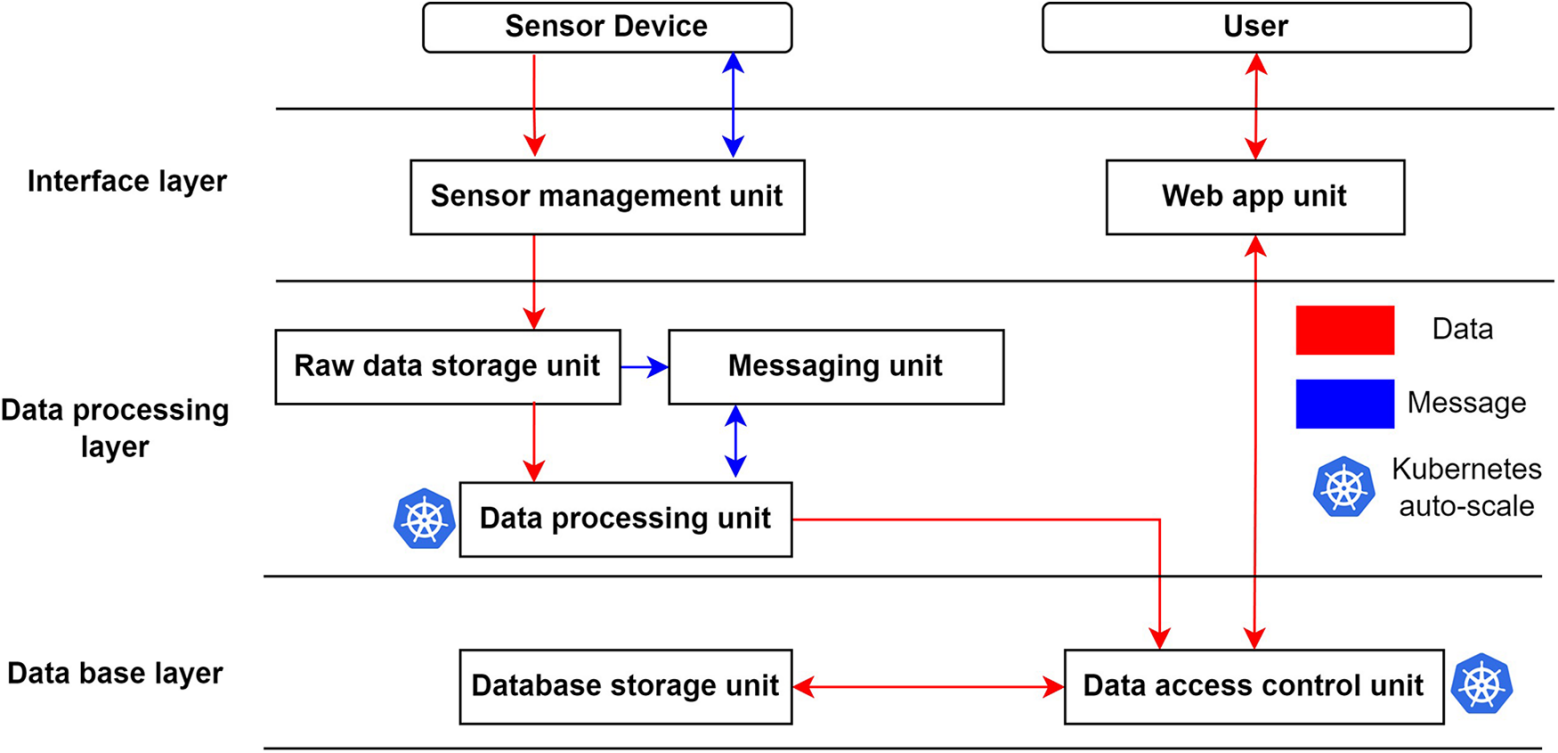
Kubernetes resource management with 2 types of autoscaler. The cluster autoscaler manages the scaling of underlying virtual machines (nodes) while the horizontal pod autoscaler manages the scaling of running application instances (pods).

In IoT applications, several relevant Kubernetes concepts as well as their arrangement are depicted in Figure 2 (17–19):

a) **Pod:** The fundamental building block of the system is the pod, which represents the containerized application with its storage space, CPU cores, and a unique IP address within the system. Auto-scaling is achieved by adjusting the number of running pods.
b) **Node:** Nodes are representations of the underlying VM structure that provide resources for running pods. They serve as the execution environment for pods.
c) **Service:** Services act as an abstract layer to access pods. Pods with the same label can be accessed through the assigned service. This allows front-end applications to interact with pods without needing to keep track of individual pod IP addresses.
d) **Ingress:** Ingress is a Kubernetes controller responsible for handling load balancing, address mapping, reverse proxy, and HTTPS connections that verify the authenticity of both the client and the server. It enables external access to services within the cluster.
e) **Deployment:** Deployments hold the specifications for the desired running state of a group of pods. This desired state includes various computing resource utilization thresholds, such as min/max CPU, memory usage, running instances, and autoscale state. Kubernetes uses this information to scale the system up or down accordingly.
f) **Pod Autoscaler:** This autoscaler adjusts the number of running pods based on the resource usage of the underlying hardware. Key parameters for this autoscaler include the time interval between adjustments, minimum and maximum number of running pods, and resource thresholds for scaling up or down. These thresholds are typically defined in terms of resource utilization percentages, such as min/max CPU, GPU, and memory usage.
g) **Cluster Autoscaler:** When the pod autoscaler determines that the required number of running pods exceeds the capacity of the underlying hardware, the cluster autoscaler steps in to address this demand by increasing the underlying hardware capacity. This is achieved by adding more computing nodes to the cluster. Critical parameters for the cluster autoscaler include resource thresholds for Kubernetes, which determine when the system should be scaled up or down in terms of minimum and maximum total nodes, CPU, GPU, and memory. Additionally, the minimum time interval between each scale-up or scale-down operation is an important parameter that prevents the system from erratically adjusting to unpredictable workloads. It is essential to set these parameters higher than those of the pod autoscaler to avoid conflicts between the two types of autoscaling.

### 2.7 Cloud services

Cloud services enable users to run applications on remotely hosted computing resources. Leveraging the extensive cloud resources managed by cloud providers alleviates developers from the technical responsibilities associated with maintaining underlying hardware and network connectivity (2). There are three primary categories of cloud service models: Software as a Service (SaaS), Platform as a Service (PaaS), and Infrastructure as a Service (IaaS). PaaS, which includes services like database and web application hosting, is maintained and managed by cloud providers. Existing services running on local servers can be easily migrated to PaaS and scaled up to meet growing demands, if necessary. However, the control over an application on a PaaS is limited to the features and functionalities provided by the cloud provider. On the other hand, IaaS grants complete control over applications and their environments, allowing for full control over the operating system and the underlying virtual machines (VMs). However, this control comes with the responsibility of managing the cloud resources, shifting the burden from the cloud providers to developers.

In our proposed architecture, we utilize a combination of PaaS services and IaaS VMs. PaaS services handle tasks such as web application hosting, load balancing, IoT gateway management, storage, and database management. On the other hand, IaaS VMs are responsible for data processing and access control to the database. Major cloud providers like Azure and AWS also offer integrated services to add extra capability to the previous modules such as monitoring and role-based access control. Our architecture can be deployed within minutes from the administrative portal, spanning multiple “operating regions”. Operating regions refer to the infrastructures on which the cloud platforms themselves are hosted, and they are situated in various regions within the United States or any host country. We provide examples of Azure and AWS cloud service modules used in our proposed architecture throughout the paper in Sections 4–6. At the time of writing, Google Cloud had retired its IoT Core component. While Azure and AWS account for the majority of the cloud market share at 80% (20), other providers, such as IBM and Oracle, are more focused on large enterprises (21).

## 3 System architecture

In our proposed architecture, we prioritize three key design considerations: ease of deployment, security, and scalability. Leveraging cloud services enables us to incorporate features like load balancing, autoscaling, role-based access control, backup, and duplication, which greatly simplify and facilitate the deployment of our IoT infrastructure. Moreover, adherence to HIPAA guidelines is a fundamental aspect of our healthcare infrastructure design. Throughout the paper, we will specify if a framework or software is open source. If not mentioned, the solution is proprietary.

**Figure 3:**
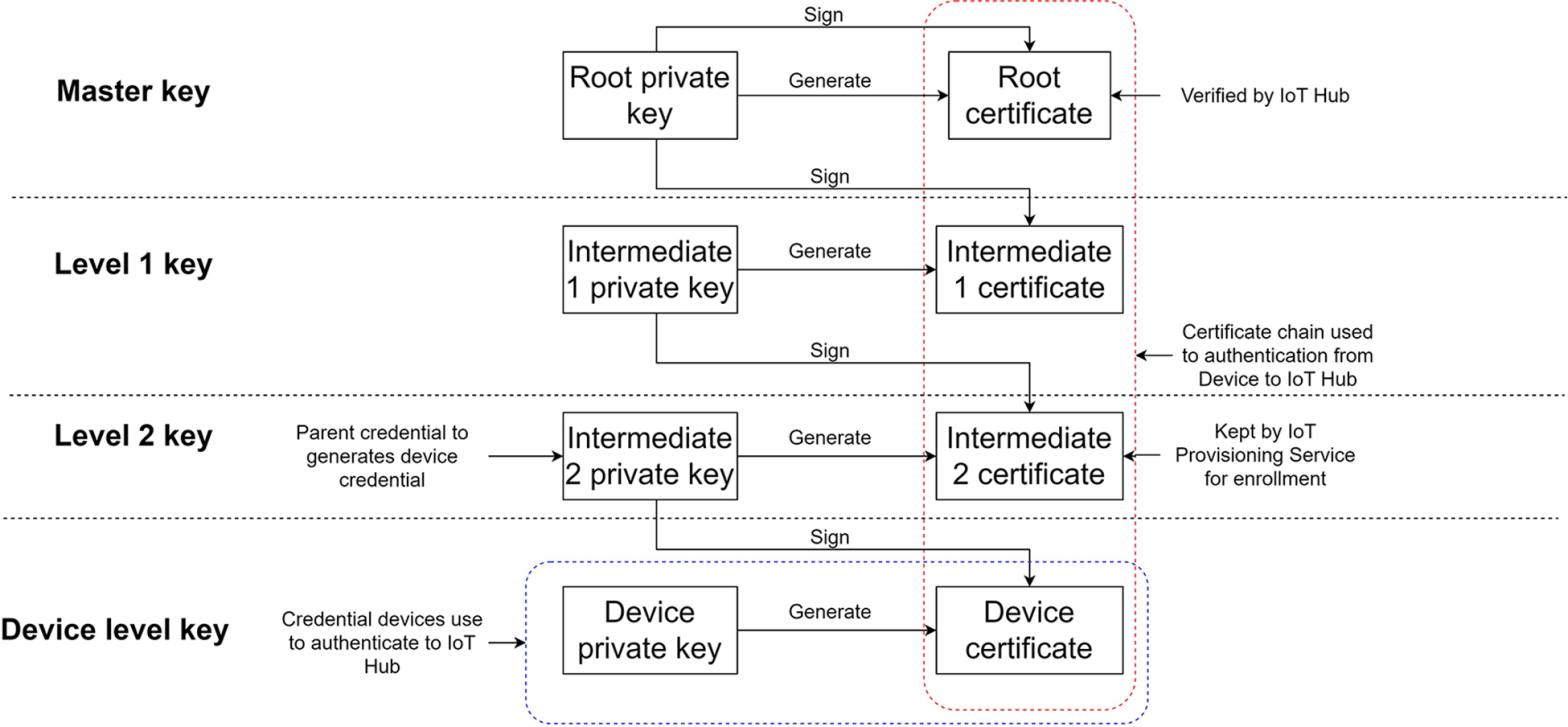
Overview of proposed system architecture. The data from the sensor network (sensor device) will flow first to the interface layer for authentication, next to the data processing layer for processing, and finally to the database layer for permanent storage. A user can access this data through the web app unit. Arrows in the figure indicate the flow of data (red) and communication messages (blue).

The proposed IoT architecture comprises three main layers, as depicted in Figure 3.

### 3.1 Interface layer

This layer facilitates communication and data transfer between the cloud services, sensor network, and users. It ensures secure isolation for the underlying system, safeguarding the integrity and confidentiality of the data.

### 3.2 Data processing layer

The primary purpose of this layer is to process the data uploaded by the sensor network and store it in the database. The raw data storage unit buffers the data received from the sensor management unit. The messaging unit facilitates communication between the buffer and the data processing unit, while also recording file processing statuses. The data processing unit leverages Kubernetes’ auto-scale feature, allowing it to dynamically adjust its computing capacity to handle fluctuating data upload volumes from the sensors.

### 3.3 Database layer

The database layer in our proposed architecture plays a crucial role in storing the processed data for efficient data retrieval and analysis. It is designed to support fast access to the data, enabling seamless data analysis operations. Additionally, the database layer handles data access authorization through an API that implements role-based authentication mechanisms. This ensures that only authorized users and other data processing layers can access the data stored within the database. Similar to the data processing unit, the data access control unit utilizes Kubernetes’ auto-scale feature to adapt its capacity based on the fluctuating data request demands. Furthermore, to enhance the security of the system, the database layer incorporates NGINX (22) as a reverse proxy. This reverse proxy setup ensures that there is no direct communication between users and any of the components within the database layer, providing an additional layer of protection against unauthorized access attempts or potential security vulnerabilities.

## 4 Interface layer

The Interface Layer is designed to facilitate interactions between sensors, users, and the cloud platform without exposing the underlying structure of the cloud services. It comprises two primary components, the Sensor Management Unit and the Web App Unit, as depicted in Figure 3. The Sensor Management Unit is responsible for managing all communication between the sensors and the cloud. This includes tasks such as device provisioning, data uploading from sensors, monitoring sensor activity, and performing updates as required. On the other hand, the Web App Unit serves as the interface for users to interact with the cloud services. This component handles tasks such as data visualization and administrative functions, enabling users to effectively engage with the cloud-based services.

### 4.1 Sensor management unit

The Sensor Management Unit serves as the intermediary for all interactions between registered sensor devices and the cloud infrastructure. This unit primarily facilitates three key types of interactions: (1) provisioning new devices, (2) sensors’ data upload, and (3) monitoring and updates. To provide context, Table 1 presents a sample of sensor types presently employed in remote healthcare applications.

**Table 1:** Example of hardware sensors used in the proposed remote patient monitoring infrastructure.

Measurement	Device/sensor	Frequency	Derived metrics
Indoor localization	BLE beacons	Hourly upload	% time in bedroom, bathroom, kitchen, other areas,% time outside of home
Outdoor movement	Absence of Beacons	Hourly upload	% time outside of home
Motion (accelerometer, gyroscope)	Smartwatch	constant monitoring, hourly upload	Time spent in different positions (e.g., laying done, standing, sitting) and activities (walking); average walking speed (e.g., time to cross 3 meters); total steps, total active time; total estimated energy expenditure,
Heartrate	Smartwatch	Hourly	Hourly averaged heartrate
Blood oxygenation	Pulse oximeter	Hourly	SpO_2_
Temperature	IR thermometer	Hourly	Temperature, changes in temperature throughout day

#### 4.1.1 Provisioning new devices

The X.509 certificate (23) plays a fundamental role in facilitating authentication between registered sensors and the cloud infrastructure. This widely accepted public digital key certificate standard serves as the basis for establishing data ownership within cloud platforms. The X.509 standard encompasses essential features, including the certificate chain and certificate path validation algorithm. The certificate chain refers to the hierarchical structure where child certificates are generated and signed by parent certificates, forming a chain. The topmost certificate in the chain is the root certificate, which is self-signed. The X.509 standard incorporates an algorithm that enables the verification of whether a given certificate, let's say certificate D, belongs to a specific certificate chain (A to B to C to D).

**Figure 4:**
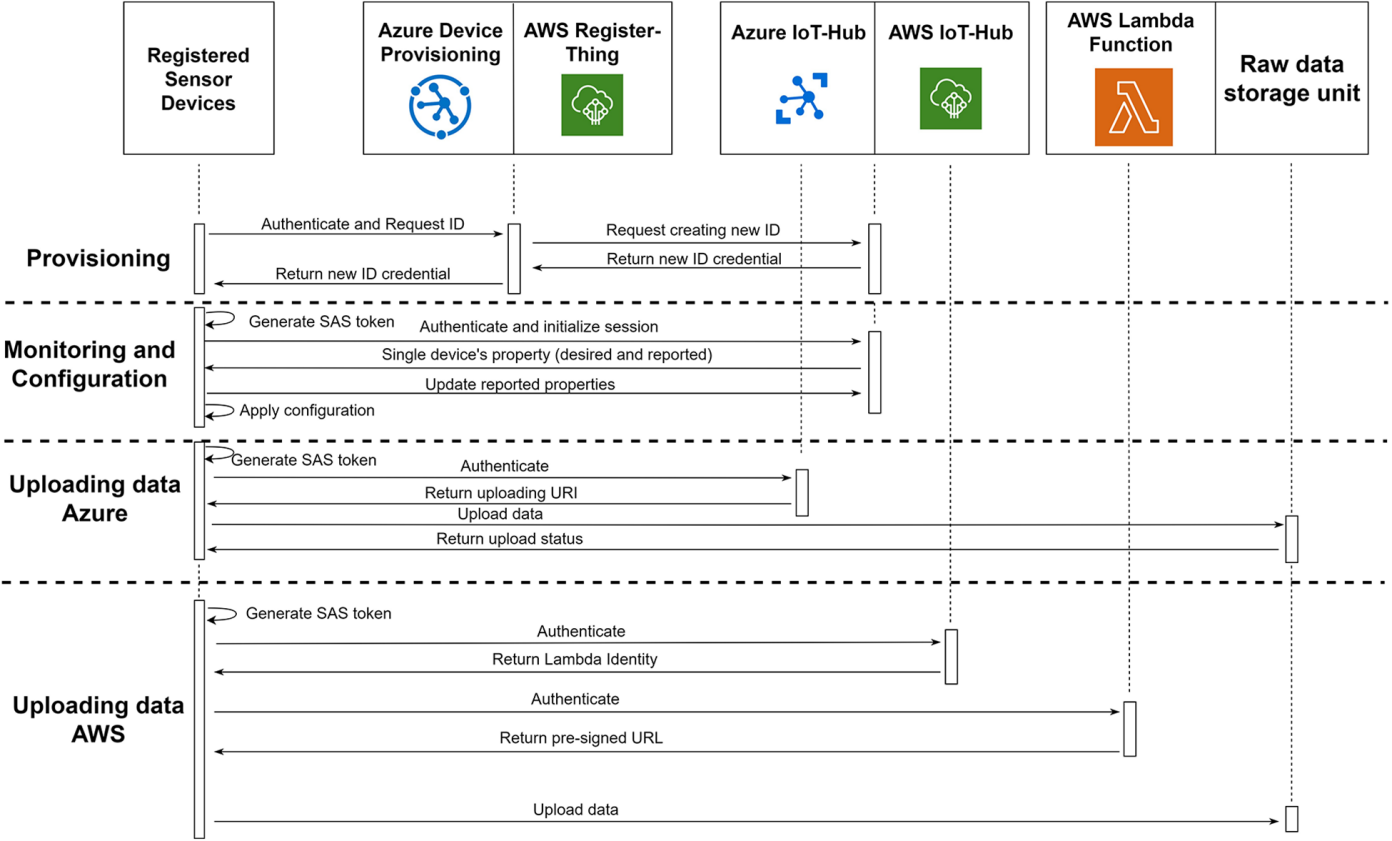
X. 509 Certificate chain setup for IoT Hub provisioning. All the registered devices will be provided with a device level key, and the certificate will be signed by the level 2 key.

In the system architecture depicted in Figure 4, the certificate chain setup is registered with the IoT Hub. The root key, known as the master key, is generated by the developer and serves as the starting point of the certificate chain. The level 2 key, on the other hand, is utilized for device enrollment, allowing the flexibility of replacing the enrollment certificate without modifying the root key. Each registered device in the system receives a certificate signed by the level 2 key, which establishes the connection to the registered certificate chain.

**Figure 5:**
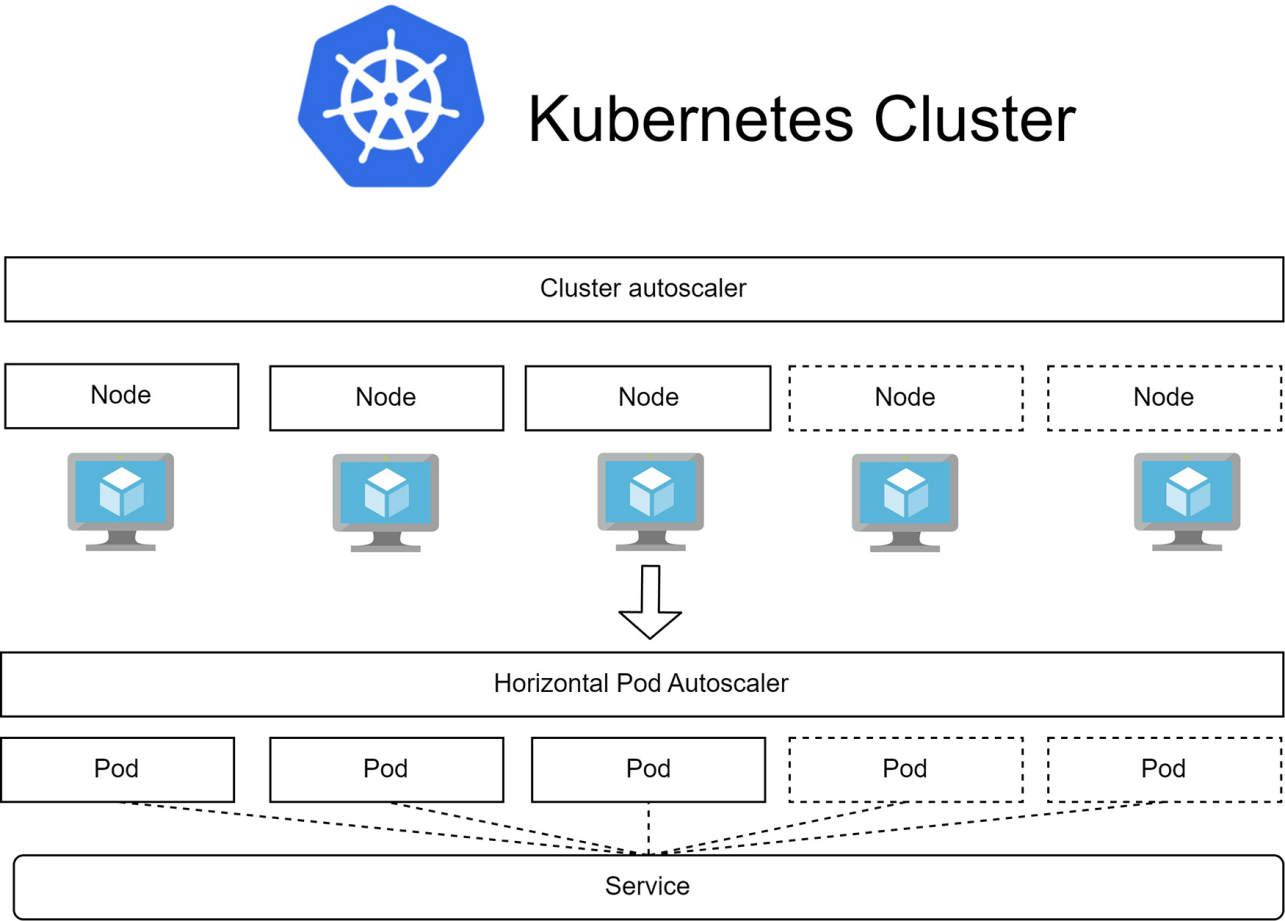
Communication between registered sensor devices with sensor management unit in both platforms for provisioning, monitoring, configuration and uploading data.

For devices to establish direct communication with the cloud, they need to possess the X.509 certificate that corresponds to the registered certificate chain in the IoT hub. This certificate serves as the authentication credential. During the initialization phase, the device utilizes this certificate to authenticate itself to either the Device Provisioning Services (DPS) on Azure or the RegisterThing (RT) on AWS, employing a secure protocol such as HTTPS or AMQPS. Upon successful authentication, DPS/RT generates a unique device identifier, credential, and initial configuration parameters. Subsequently, DPS/RT requests the IoT-Hub to register the new identity within the system. Once the registration process is complete, DPS/RT forwards the new identity to the device. The device then utilizes this identity to communicate with the IoT-Hub. The provisioning segment in Figure 5 illustrates this process.

#### 4.1.2 Sensors’ data upload

Below, we discuss the Azure and AWS data uploading process separately due to the difference in how their respective IoT-Hub modules work. The code to handle these processes is provided as software development kit (SDK) from Azure and AWS to be incorporated into the sensor device firmware.

##### 4.1.2.1 Azure

When a registered device intends to upload data to the IoT-Hub, it utilizes its credentials to generate a shared access signature (SAS) token, which has been pre-registered at the IoT-Hub with a predefined expiration time. This SAS token serves as the device's authentication mechanism with the IoT-Hub. Once authenticated, the device proceeds to upload the data to the IoT-Hub via the HTTPS protocol. Upon receiving the new data, the IoT-Hub forwards it to the raw data storage unit and sends a success notification back to the device. This implementation ensures that all registered devices upload files to the cloud through the IoT-Hub without needing to know the actual endpoint. This separation between the device and the cloud enables the IoT-Hub to monitor all sensor data traffic entering the system. For faster data upload, the device has the capability to upload multiple files concurrently. However, it is important to note that each connected device has a maximum limit of 10 concurrent file uploads imposed by the IoT-Hub. Additionally, each IoT-Hub module can handle a maximum of 10,000 concurrent file upload connections at any given time (24). If there is a requirement for more concurrent file upload connections, it is possible to divide the registered device into multiple IoT-Hub instances linked to the same raw storage unit. Figure 5 illustrates the entire process of data uploading in Azure.

##### 4.1.2.2 AWS

In the AWS platform, there are limitations on the file size for direct data upload integration between the IoT and storage services, with a maximum file size of 128 KB, which may be insufficient for many applications. To overcome this limitation, we propose the use of an additional Lambda Function module that provides devices with a temporary direct upload link known as a pre-signed URL. This pre-signed URL allows for direct data uploads without any file size constraints. Each pre-signed URL is valid for a specific file and expires after a predefined interval allowed it to serve a similar purpose as SAS token on Azure side.

To upload data using the pre-signed URL, the registered device utilizes its credentials to authenticate with the IoT-Hub. The device retrieves its “IoT Device Management” property from the IoT-Hub, which contains the URL for communication with the Lambda Function module. The device then uses the same credentials to authenticate with the Lambda Function module and sends the desired file's name. The Lambda Function generates a pre-signed URL that is unique to the specified file name. The device can subsequently use this pre-signed URL to directly upload data to the raw data storage unit, bypassing any limitations on file sizes. The entire process of data uploading in AWS, including the usage of pre-signed URLs and the involvement of the Lambda Function module, is depicted in Figure 5.

#### 4.1.3 Monitoring and update

In the IoT-Hub, each registered device has two types of configuration properties: desired and reported. These properties, presented in JSON format for easy readability and editing, serve different purposes. Desired properties are parameters that can only be modified by developers and are typically utilized for remote configuration. Reported properties, on the other hand, can be overwritten by the registered device, enabling the device to update its status to the IoT-Hub.

To retrieve and update these configuration properties, the device must first authenticate itself with the IoT-Hub using its credentials. Subsequently, the device can utilize the provided methods within the cloud platform's SDK to access and modify the desired and reported properties. The process of updating device properties is illustrated in the “Monitoring and update” Section 4.1.3 of Figure 5.

These desired properties can be used to implement a remote firmware update procedure. The server first includes the desired version number and a download link pointing to the new firmware. Then, the registered device retrieves the desired property from the server and compares the current version number with the desired version number. If the desired version number is higher than the device's current version, the device proceeds to download the new firmware from the provided link in the property and performs the necessary update.

We currently use the desired properties for sensor devices’ firmware updates, adjusting survey time, which survey to used and how often survey should be displayed for example.

### 4.2 Visualization: Web app unit

The web app unit serves as a platform for authorized users to access patient data and visualize inferences made from raw data. It incorporates several crucial functionalities, including load balancing to evenly distribute the server workload, establishment of secure connections using the HTTPS protocol for data requests between clients and servers, and scalable computing resources to handle fluctuations in the number of connections. It can also serve as a portal for user registration including doctors, nurses, patients or clinical professionals. The administrative of the RBAC system will determine each user's accessibility as discussed in 6.1.2. Our webapp is made with AngularJS, the open-source front-end webapp framework for interactive websites. The webapp is then converted into docker image to be deployed as described below.

#### 4.2.1 Azure visualization

For hosting the web app on the Azure platform, Azure App Services is a suitable cloud module. It provides TLS certificates, public IP addresses, configurable auto-scaling (with minimum and maximum number of web app server instances), and load balancing across multiple running web app instances. Deploying the web app can be achieved by linking the code repository (e.g., GitHub, Bitbucket) of the web app to the Azure App Services. Alternatively, if the developers have containerized their web application to leverage the auto-scaling features of Kubernetes, they can link the container registry (e.g., Docker Hub, Azure Container Registry) to the web app cloud services for an alternative deployment method. The Kubernetes auto-scaling feature will be discussed in Section 5.3.1.

Additionally, another option for providing a web app interface is by utilizing the Power BI (Business Intelligence) module. Power BI is a data visualization module with a built-in graphical user interface (GUI) that allows users, even those with limited programming experience, to generate analytical graphs. The GUI offers a comprehensive set of support functionalities, such as importing data from various sources (databases, Excel, web hooks, etc.), performing data calculations using Excel-like command logic, and creating graphs using provided graph models as well as third-party graph model extensions. However, the ability to customize the calculations and graphs is very limited to what the platform provides, contrary to Azure App services. In cases where the underlying data source to be updated and visualized regularly exceeds 10 GB, we propose using Azure Analysis Services (AAS) in conjunction with Power BI. AAS is a data aggregation module capable of collecting data from multiple sources and processing them to produce tables ready for visualization. This enables AAS to act as a cache for Power BI, facilitating the loading of data into the visualization model. It is worth noting that Power BI is costly if the data synchronization needs to happen many times a day. Normally Power BI is suitable if you need to visualize data after a day of collecting information. Using Power BI might be challenging in healthcare applications where real-time or near real-time and continuous monitoring of data is required.

#### 4.2.2 AWS visualization

On the AWS platform, AWS Elastic Beanstalk is an appropriate cloud module for hosting the web app. It also provides TLS certificates, public IP addresses, configurable auto-scaling (with minimum and maximum number of web app server instances), and load balancing capabilities across multiple running web app instances. Similar to Azure, the web app can be deployed by providing the link to the code repository of the web app to AWS Elastic Beanstalk. Containerized web applications can also be linked to the cloud services through a container registry (e.g., Docker Hub, AWS Container Registry).

#### 4.2.3 User registration in bulk using the web app unit via azure and AWS

In scenarios involving the need to register a large number of patients simultaneously, Microsoft Entra ID and AWS Cognito can be integrated with the web app modules for authorizing users and managing user registrations in bulk. Registered users can be assigned to group role identities, allowing for granular control over resource access levels and password policies for each group. Both Microsoft Entra ID and AWS Cognito comply with HIPAA regulations, utilizing OAuth 2.0 standard-based authentication and offering built-in support for multi-factor authentication. These modules can be integrated into Azure App Services and AWS Elastic Beanstalk, respectively, using Azure and AWS software development kits (SDKs).

## 5 Data processing layer

Our proposed data processing layer, illustrated in Figure 1, comprises three essential components: (1) raw data storage unit, (2) messaging unit, and (3) data processing unit. The raw data storage unit serves as a cloud service, playing a crucial role in buffering the bursts of data sent from the sensor network at any given time. Besides its primary function as a buffer, the low storage cost offered by cloud platforms enables the unit to store raw data long-term directly in the cloud. This capability grants our system the flexibility to reprocess historical data if needed, particularly when refining or altering the analytics algorithms. The messaging unit is responsible for notifying the data processing unit whenever a new file is uploaded. Additionally, it records the status of successful and failed file processing operations. The data processing unit takes the new file, which is notified by the messaging unit, from the raw data storage. Subsequently, it converts the raw data into a queryable format before forwarding it to the database layer.

### 5.1 Raw data storage unit

The primary role of this unit is to serve as a storage repository for the raw data uploaded from the sensor network. Although there are many open-source data warehouse solutions such as Apache Hadoop or Databricks, the complexity of setting up a storage system that is scalable, secured and affordable is quite daunting. Therefore, we opt to use Azure Blob Storage and Amazon S3 cloud modules used for this purpose instead. These storage modules consist of smaller storage units known as containers for Azure Blob Storage and buckets for Amazon S3. When files are uploaded to the linked IoT Hub, they are automatically forwarded to the designated container/bucket within the raw data storage unit. Upon successful upload, the uploading devices receive a confirmation message. In addition to storing sensor data, we propose utilizing a section of the raw data storage unit to store configuration files or software update patches for sensor edge devices. This can be achieved by creating another container/bucket or storage account to maintain separation between cloud services. The link to download the software or configuration files will be stored in the properties of the corresponding devices on the IoT-Hub. This approach facilitates efficient management and deployment of software updates and configurations for the sensor edge devices.

#### 5.1.1 Security

We suggest the following measures to enhance the security of the Raw Data Storage Unit:

a) **Use Shared Access Keys:** Instead of using the root access keys, which have extensive privileges, provide applications with shared access keys. These keys can be customized with specific configurations, including allowed actions, access protocols, allowed IP addresses, and expiration time. By using shared access keys, you can limit the permissions granted to each application, reducing potential risks.
b) **Implement Network Access Controls:** Set up a firewall to allow access to the storage account only from whitelisted IP addresses and virtual networks. This network access control ensures that only authorized entities can interact with the storage unit, enhancing overall security and controlling traffic flow on the server-side.
c) **Utilize Customer-Managed Encryption Keys:** By default, the data in the storage account is encrypted using Microsoft-managed keys or AWS-manage keys. However, to further enhance data security, developers can opt to use their own uploaded encryption keys. This provides an additional layer of control and protection over sensitive data.
d) **Implement Logging and Monitoring:** Create a dedicated logging container/bucket to record and store every operation in the storage modules. This comprehensive logging is essential for monitoring the system's activity, ensuring security, and meeting compliance requirements, such as HIPAA.

By implementing these security measures, the Raw Data Storage Unit can provide a higher level of protection for sensitive data, minimize potential vulnerabilities, and ensure the confidentiality and integrity of the stored information. We enable all these measures in the configuration options for Azure Blob Storage and AWS S3.

#### 5.1.2 Data availability

We recommend the following strategies to improve data availability in case of cloud raw data storage service outage:

a) **Data Replication:** Store multiple copies of the data to protect against outages. The common practice is to maintain three copies of the data in the main region of deployment (local redundancy) and an additional copy in a separate region as a backup (geo redundancy). In AWS S3, local redundancy is enabled by default, and geo redundancy can be achieved by enabling the Cross-Region Replication feature in the storage configuration. In Azure Blob Storage, data replication features are not enabled by default, but both local and geo redundancy can be achieved by enabling the Geo-redundant storage feature in the storage configuration.
b) **Regular Backups:** Implement a regular backup strategy to periodically create snapshots or backups of the data. These backups should be stored in a different location or region from the primary data storage.
c) **Load Balancing:** Multiple storage modules can be used concurrently to handle large number of user base. Utilize load balancing techniques to distribute incoming data requests across multiple storage instances or regions. This can help prevent overloading of a single storage unit and improve overall data availability. In case of an outage, the latest backup can be used to restore the data.

By implementing these suggestions, organizations can enhance the data availability and resilience of their cloud raw data storage service. This ensures that critical data remains accessible even in the face of unexpected outages or disruptions. We enable local redundancy, daily backup for both Azure Blob Storage and AWS S3 modules. We did not use the load balancing solution as the base ingress bandwidth is already very high for us (60 and 100 gigabit per second for Azure and AWS respectively).

### 5.2 Messaging unit

The role of this unit is to facilitate communication between the raw data storage unit and the data processing unit by notifying the latter of newly uploaded files. When a file is uploaded to the Azure Blob Storage/Amazon S3, an auto-generated message containing relevant information about the uploaded file is created. This message is then forwarded to a messaging service, which in turn notifies the data processing module to fetch the new file(s) for processing. Both Azure and AWS offer two main types of messaging modules: pull and push.

In the pull module, the receiver takes the initiative to pull new messages, thereby placing message flow control on the receiver side. Cloud messaging modules serve as message buffers, holding messages until a receiver requests them. Once a receiver completes processing a message, it requests the cloud message buffer to delete the message from the queue and request the next one. To avoid multiple receivers processing the same message, the requested message is made visible to only one receiver at a time. Azure and AWS offer pull-type messaging services named Azure Queue Storage and AWS Simple Queue Storage, respectively. These services facilitate efficient and reliable message handling, making them suitable for various applications that require reliable message delivery and processing.

Conversely, the push module operates on a publisher and subscriber model. New messages are stored at the publisher's side within a specified topic, and multiple subscribers maintain connections to the topic to receive new messages. The publisher pushes the message from the topic to active subscribers, enabling the publisher to control the message delivery rate. This type of messaging is suitable for scenarios where the same message needs to be distributed to multiple receivers, such as news outlets distributing articles to subscribers. Azure and AWS offer push-type messaging services called Azure Service Bus and AWS Simple Notification Service.

In our proposed system, we opt for the pull module since each generated message from the messaging unit should be received only once by the data processing unit. Whenever a new file is uploaded to the raw data storage unit, a message containing the file's address is generated and stored in the pending queue. We recommend using two additional message queues to keep track of successful and failed processing attempts. The failed queue can be revisited once any possible errors are rectified, enabling the reprocessing of files.

### 5.3 Data processing unit

This unit listens for new messages that indicate the arrival of raw data. Upon detection, it proceeds to download and process the raw data into query format, subsequently making API requests to the database layer to upload the processed data.

To build cloud applications like data processing or data access control, we recommend utilizing the Spring Boot framework. This open-source Java-based framework simplifies development by providing “starters”, which are pre-configured templates for common server services such as database querying, web services for API mapping and role-based authentication, messaging services, cloud integration, and thread-scheduling management. Developers can customize these starters to meet their specific requirements, streamlining the development process. The final application is containerized into an image file, facilitating deployment on any platform that supports containers. Docker is the suggested containerization platform, given its popularity, strong support from the development community, and wide adoption on various cloud platforms (25).

Regarding running applications on cloud platforms, we propose using container services, referred to as Container Services in Azure and Elastic Container Service in AWS. These services host containerized applications, enabling portability. By defining configuration parameters like CPU size and VM storage through the administrative portal, the cloud container can be deployed within minutes. The application can then be scaled in two ways: scaling up by upgrading the underlying hardware's capacity (increasing CPU and RAM) or scaling out by adding more units of the same underlying VM. For applications requiring dynamic runtime scaling (a key purpose of our architecture), scaling out by adding more VMs is the more suitable method since adding more VM instances does not disrupt the VMs currently running. This can be done manually by adding more VMs to the cloud Container Service.

However, the Container Services and Elastic Container Service do not offer automatic scaling based on workload demand. Therefore, Kubernetes Service and Elastic Kubernetes Service (in Azure and AWS, respectively) are usually being used in conjunction to act as container management units that allow for such automatic scaling of applications. By integrating these services, the cloud architecture can efficiently manage the scaling of applications based on demand.

#### 5.3.1 Kubernetes system implementation

**Figure 6:**
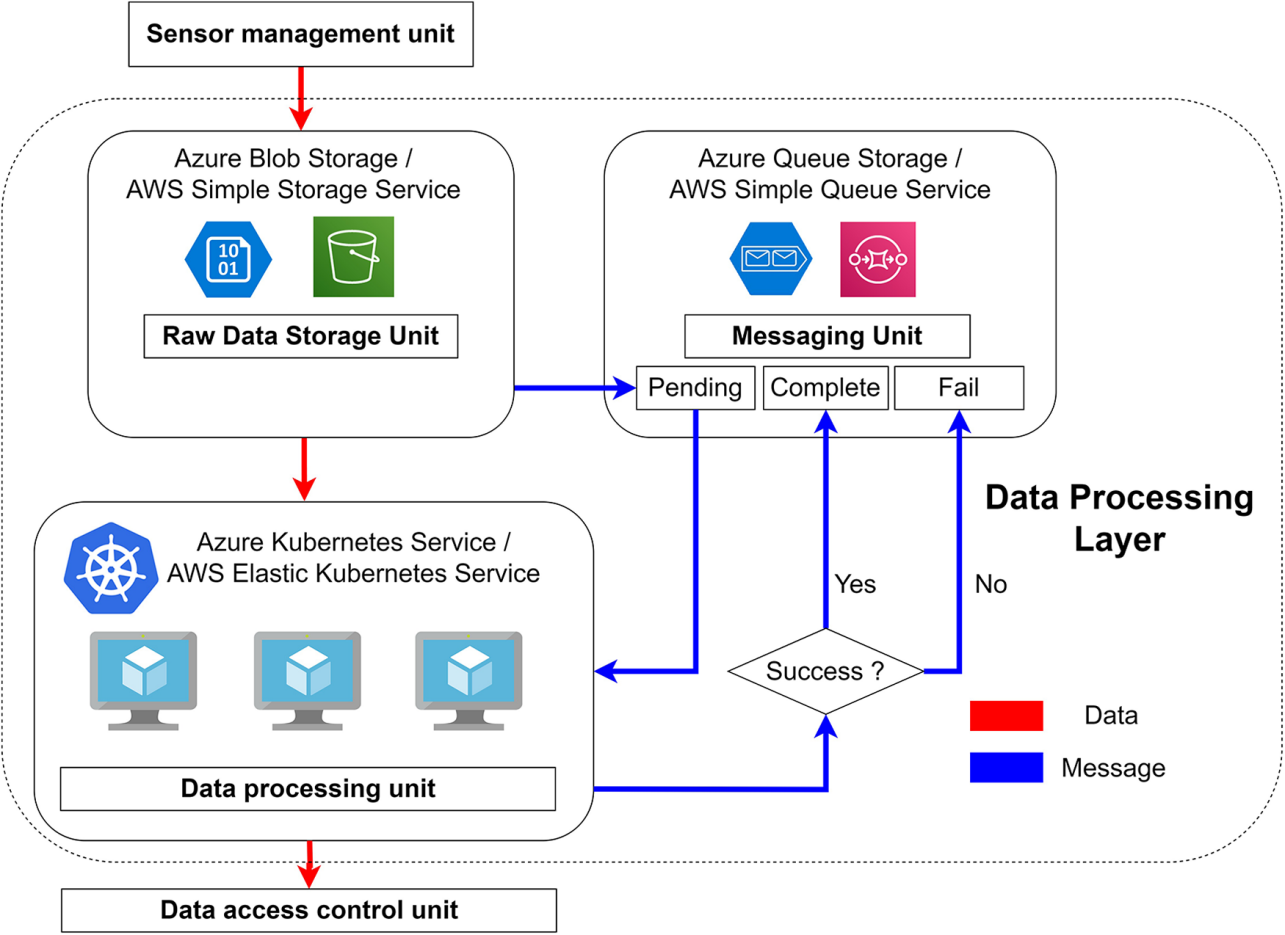
The highlighted block inside the dashed line illustrates the data processing layer. The sensor management unit uploads data to the raw data storage unit where a message is sent to the “pending” queue in the messaging unit with the path of the new file. The Kubernetes service listens to the new message, downloads the file from the storage service using the content of the message, processes it, and sends the results to the data access control unit. The received message will be redirected to either the “complete” or “fail” queue, depending on the results.

Figure 6 illustrates how we integrated the Kubernetes system into our data processing layer. The data processing application is deployed as an image file to either the Container Services in Azure or the Elastic Container Service in AWS. These cloud services are then connected to the Kubernetes Service in Azure or the Elastic Kubernetes Service in AWS, respectively. In either cloud platform, the Kubernetes service generates and manages multiple pods, with each pod running an instance of the data processing application.

The data processing application continuously listens for messages from the “pending queue” of the messaging unit. These messages contain the file path necessary to download the newly uploaded file from the raw data storage unit. Upon receiving the message, the application fetches the uploaded file from the raw data storage unit using the information provided and processes it accordingly. If the file is successfully processed, the application sends the original message to the “complete queue” and uploads the processed file to the data access control unit of the database layer. On the other hand, if processing fails, the message is sent to the “fail queue” for further handling. The “complete queue” allows for reprocessing previously completed data in the event of algorithm changes, while the “fail queue” enables the system to reprocess failed files.

To ensure security, it is essential to implement access control mechanisms across various modules of the cloud infrastructure. For instance, the data processing application should be equipped with the necessary credentials to access the raw data storage unit, the messaging unit, and the data access control unit. Implementing authentication between all components of the system is considered a good security practice, safeguarding against unauthorized access and potential security breaches.

Overall, the integration of the Kubernetes system in our data processing layer enhances efficiency, scalability, and reliability, while the implementation of access control measures bolsters the security of the entire cloud infrastructure.

## 6 Database layer

**Figure 7:**
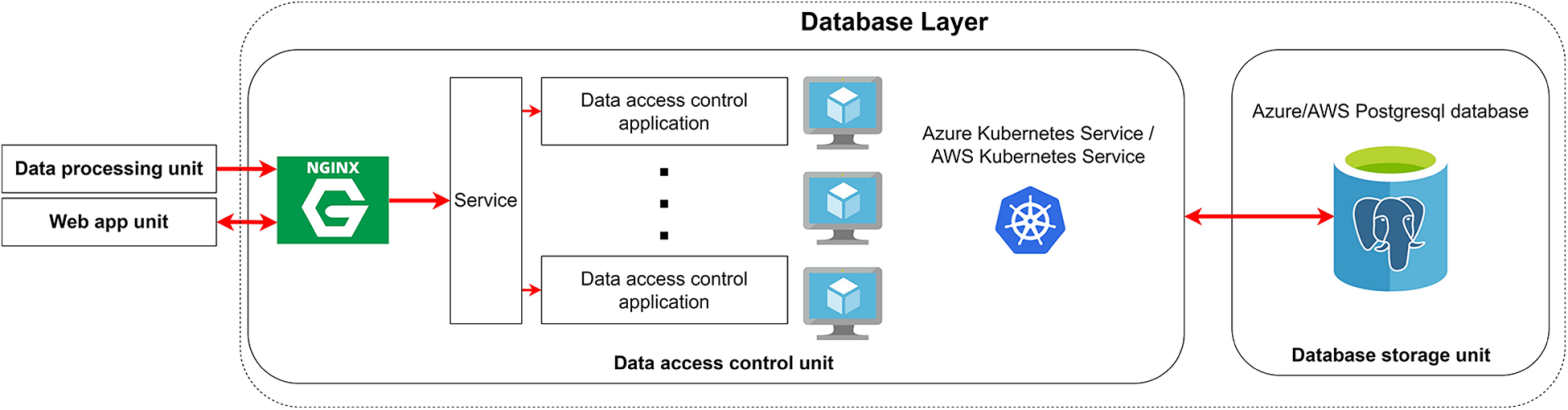
Database layer structure. The Kubernetes services will run multiple instances of data access control applications that communicate directly to the PostgreSQL database and share the same API call to handle the incoming requests. These requests that come from the data processing unit or web app unit are funneled through Nginx Ingress Controller to distribute the load evenly and isolate the data access control application through the reverse proxy.

The Database Layer's primary objective is to store data in a query format accessible through API requests from multiple clients, including users and other system layers, with proper role-based authentication. Establishing secure connections and load balancing the concurrent data access requests from clients are essential functionalities for this layer. It comprises two major components: a) the data access unit, responsible for handling secure connections with load balancing capabilities, and b) the database storage unit, tasked with storing the data. Figure 7 provides a visual representation of the data flow in the database layer.

### 6.1 Data access control unit

#### 6.1.1 Load balancing

The load balancing component in the Database Layer serves two primary functions: maintaining a secure connection between the client and the server through the ingress control web interface and evenly distributing client requests among multiple data access control applications through load balancing abstraction.

The public-facing ingress control interface employs the Nginx. Nginx is an open-source web server software that specialized in reverse proxy and load balancing. We use Nginx software to act as a reverse proxy only. Nginx establishes Transport Layer Security (TLS) connections and facilitates secure communication between the public internet and the database layer components. TLS adds an extra layer of security on top of the traditional Hypertext Transfer Protocol (HTTP) by verifying the server's authenticity through a digital certificate and encrypting all data transferred between the client and the server (26). The combination of HTTP and TLS forms the Hypertext Transfer Protocol Secure (HTTPS), a foundational protocol for modern internet communication. The digital certificate, obtained from third-party certificate authorities or cloud providers, is supplied to Nginx to enable the TLS layer. The reverse proxy aspect of Nginx ensures that the underlying server remains hidden from clients, acting as an intermediary between clients and servers. All client requests are directed to Nginx ingress control before being forwarded to the load balancing abstraction, guaranteeing that clients do not directly interact with the server, thus enhancing the overall system's security.

The load balancing abstraction, facilitated by Kubernetes, ensures even distribution of client requests among multiple instances of data access control applications. Identical pods running the data access control application are grouped into a single service dispatcher. When a request is received, the service dispatcher assigns the request to an available pod in a round-robin fashion. An advantage of this load balancing approach is that it automatically reconfigures routing distribution when pods scale up/down or crash, eliminating the need for manual intervention.

#### 6.1.2 Database access control using application programming interface (API)

For rapid development, we again employed the Spring Boot framework. Within the Spring Boot framework, the Tomcat module handles web server hosting by assigning network ports to respond to client requests. Additionally, the Spring Hateoas module manages API request routing, matching user requests to the appropriate server responder. Furthermore, the Hibernate API formats the database table into a Java class template. For instance, when a client requests patient data, the API fetches the corresponding information from the patient table and formats it according to the Java class template to construct a response for the client. To manage Role-Based Access Control (RBAC), the Spring Security module is used in the system. Three types of roles are assigned to each user, determining the scope of authorized data viewable by users. The patient role permits access only to data that belongs to the patients themselves, while the doctor role grants access to data of all patients assigned to the doctor. The admin role, on the other hand, has access to all data in the system, and it can also register new patients and assign them to doctors. The API validates user requests by checking the users’ credentials against the hashed versions stored in the database. Overall, the Spring Boot framework considerably reduces the technical burden of server development by providing pre-built modules that handle various common server tasks.

Once the Spring Boot database access control application is completed, we use Docker to convert the application into an image file to make deployment in any cloud platform easier. The load balancing unit from the previous section will also be converted into an image file for deployment in similar fashion. For Azure and AWS, the entry for the deployment image file is the Azure Container Registry and Amazon Elastic Container Registry respectively. Kubernetes then utilizes this image file to generate and manage multiple instances of the data access control application, automatically scaling them up or down according to the server's requested auto-scale configuration.

### 6.2 Database storage unit

We used PostgreSQL database to store the processed data for visualization and analytics purposes. PostgreSQL is an open-source database system that organizes data in relational and key-value format. For our application, we need to migrate our existing PostgreSQL database to the cloud. There are four essential features that we are looking for in the cloud database module: availability, security, latency, and scaling.

Availability is ensured by cloud providers through automatic backup, replication, and hardware problem detection. Data duplication occurs in two additional instances, one within the same region and another in a different selected region.

For security, all data in the database is encrypted at rest and during transit via HTTPS. Role-based access control can be implemented to prevent credential leakage. The database's security can be further enhanced by configuring the network security firewall to permit access only from the registered virtual private network and IP address.

Latency and scaling are crucial factors affecting database query performance. Latency is influenced by computing resources, network speed, and query efficiency. The computing resource can be scaled by changing the number of CPU cores to handle extensive query demands. Network speed can be improved by utilizing a separate database to store high-demand content using SSD instead of HDD balances low latency with cost savings. Geo-replication and distributed regional storage can further reduce latency by minimizing the distance between requesters and the database. Additionally, query efficiency can be achieved by minimizing the number of query requests and creating a second database with a different structure for faster retrieval to cater to specific high-demand requests.

Azure and AWS offer various PostgreSQL database hosting options on their platforms. The specific one that we used are Azure Database for PostgreSQL and AWS Aurora PostgreSQL respectively.

## 7 Infrastructure setup cost

In this section, we delve into the estimated costs associated with setting up a comprehensive cloud infrastructure on both Azure and AWS platforms for the US WEST 2 region and US WEST North California region, respectively. Our chosen setup encompasses key components such as IoT services, raw data storage solutions, virtual machines, databases, and web application hosting. The goal is to provide cloud developers with valuable insights into the pricing structures of these platforms, facilitating an informed decision-making process based on their unique operational demands.

The estimated monthly cost Azure setup is for the US WEST California region encompasses a variety of essential cloud components mentioned in the previous sections. Azure IoT Hub handles communication from 10,000 devices, each transmitting one message per hour. Azure Blob Storage provides 1 TB of storage capacity, supporting up to 100,000 read/write operations monthly. Two Azure VM D2v3 instances, each with 2 cores, 8 GB RAM, CentOS OS, and 64 GB SSD storage, collectively perform 1,000 input/output operations per second (iops) monthly. The Azure PostgreSQL database, hosted on a D2Sv3 VM instance with 500 GB storage, 2 cores, and 8 GB RAM, handles up to 1,000 iops each month. The Azure App Service utilizes an S2 instance with 2 cores, 3.5 GB RAM, and 50 GB storage for application hosting.

On the AWS front, we do the setup for the US WEST North California region mirrors the functionality of its Azure counterpart. AWS IoT Core manages communication from 10,000 devices, each sending one message per hour. AWS S3 Storage provides 1 TB of storage capacity, supporting up to 100,000 read/write operations monthly. Two AWS t3.large instances, featuring 2 cores, 8 GB RAM, CentOS OS, and 64 GB SSD storage, collectively perform 1,000 iops monthly. The AWS RDS PostgreSQL database, hosted on a tdb.3.large VM instance with 500 GB storage of provisioned IOPS SSD, 2 cores, and 8 GB RAM, handles up to 1,000 iops each month. AWS Lightsail, equipped with 2 cores, 4 GB RAM, and 80 GB storage, offers a simplified and cost-effective solution for application hosting. Azure VM D2 v3 2 cores, 8 GB ram CentOS 64 GB temporary storage US WEST 2 1,000 iops.

Table 2 provides a detailed breakdown of the estimated costs for each component in the relevant cloud architecture, incorporating reduced pricing for advanced reservations applicable to VM and database components. The cloud setup in Table 2, based on pricing as of July 2024, is sufficient to process data for 200 concurrent patients, ranging from $463.05–$645.18 per month for Azure and $409.31–$567.00 per month for AWS. The lower prices reflect the discount for reserving the server for three years, compared to the more expensive on-demand system. The primary cost contributors are the VM, database, and webapp components. In summary, the AWS platform proves approximately 10% more cost-effective than the Azure platform for the entire system setup. This cost disparity is mainly attributed to the significantly lower expenses associated with hosting web applications on AWS compared to Azure. Furthermore, Azure offers more affordable database services, while AWS presents less expensive VM services. Considering the potential for a one-third reduction in costs by reserving the system for up to 3 years, developers are advised to reserve the minimum required resources, optimizing cost savings while retaining the flexibility of on-demand scalability to accommodate surges in server requests. This cost breakdown only includes the operational cost of cloud services. Other costs, such as human resources for system development and maintenance, or labor costs for distributing data collection devices (e.g., smartwatches, tablets, or other sensor devices) to patients, will vary depending on the scope and technical demands of the project.

**Table 2:** Estimated monthly cost in US dollars to set up the entire cloud architecture between azure and AWS platform recorded as of march 2024.

Cloud component	On-demand		1-year reserved		3-year reserved	
	Azure	AWS	Azure	AWS	Azure	AWS
IoT Hub	25.00	42.06	25.00	42.06	25.00	42.06
Blob Storage	22.41	29.64	22.41	29.64	22.41	29.64
2 VM	90.21 × 2	78.99 × 2	62.72 × 2	52.06 × 2	41.85 × 2	36.87 × 2
Database	271.35	317.7	214.34	278.06	185.94	244.25
Webapp	146.00	19.62	146.00	19.62	146.00	19.62
Total	645.18	567.00	533.19	473.50	463.05	409.31

## 8 System testing

As we developed our system on both Azure and AWS for our remote monitoring system, we documented some performance benchmarks. The goal is to give an example of performance change when moving between AWS and Azure for workload pipeline similar to our work. More variety of workflow and configuration are needed to properly draw a comprehensive comparison between the AWS and Azure platform. The workflow used for the test benchmark in this section is based on our previous work from the Sensing At-Risk Population study (4), where we collected data from a cohort of 110 geriatric patients at a rehabilitation center over the course of 21 days. This allows our test benchmark to more realistically represent the performance of other remote monitoring studies.

### 8.1 Data processing layer performance test

The performance test conducted in the data processing layer aimed to evaluate the impact of scaling on the speed of the cloud system's data processing unit. Additionally, the test sought to compare the performance of the system on different cloud platforms while ensuring a fair comparison by selecting VM configurations with similar hardware capacities. Table 3 presents the VM configurations and Kubernetes settings used in the performance test. The VMs from Azure and AWS were chosen, and they were connected to their respective storage and messaging services within the same region to eliminate network-related factors.

**Table 3:** Configuration of the VMs and kubernetes cluster in the azure and AWS platform.

Cloud platform	Azure	AWS
Instance name	Standard DS2 v2	m5.large
Processor	Intel Xeon E5-2673 v3 @ 2.4 GHz	Intel Xeon® Platinum 8175 @ turbo 3.1 GHz
Cores	2	2
RAM	7	8
Local storage type	SSD	Amazon Elastic Block Store
Number of nodes in cluster	5	5

The testing procedure involved simulating a data processing workflow in a healthcare application. A set of 1,000 accelerometer raw data files, with an average size of 1.5 MB and a sampling frequency of 16 Hz, was uploaded to the storage units. Subsequently, 1,000 messages were generated and sent to the message queuing unit. The data processing unit within the Kubernetes cluster listened to these messages and ran the file processing application in parallel using multiple pod instances being scaled by the Kubernetes system.

The test case scenarios were designed to examine the effect of scaling on file processing speed. In the first three test cases, the number of running pod instances was manually scaled at 2, 4, and 8, while all five nodes were available. This allowed for observing the impact of pod scaling on the file processing speed. In the fourth test case, the horizontal pod autoscaler took charge of the scaling, starting with one running pod and five nodes. This scenario provided insights into the performance when scaling was handled by the autoscaler. In the fifth test case, both the horizontal pod autoscaler and the cluster autoscaler were utilized for scaling, starting with one running pod and one node. This configuration allowed for assessing the combined effect of both autoscalers on the system's performance.

**Figure 8:**
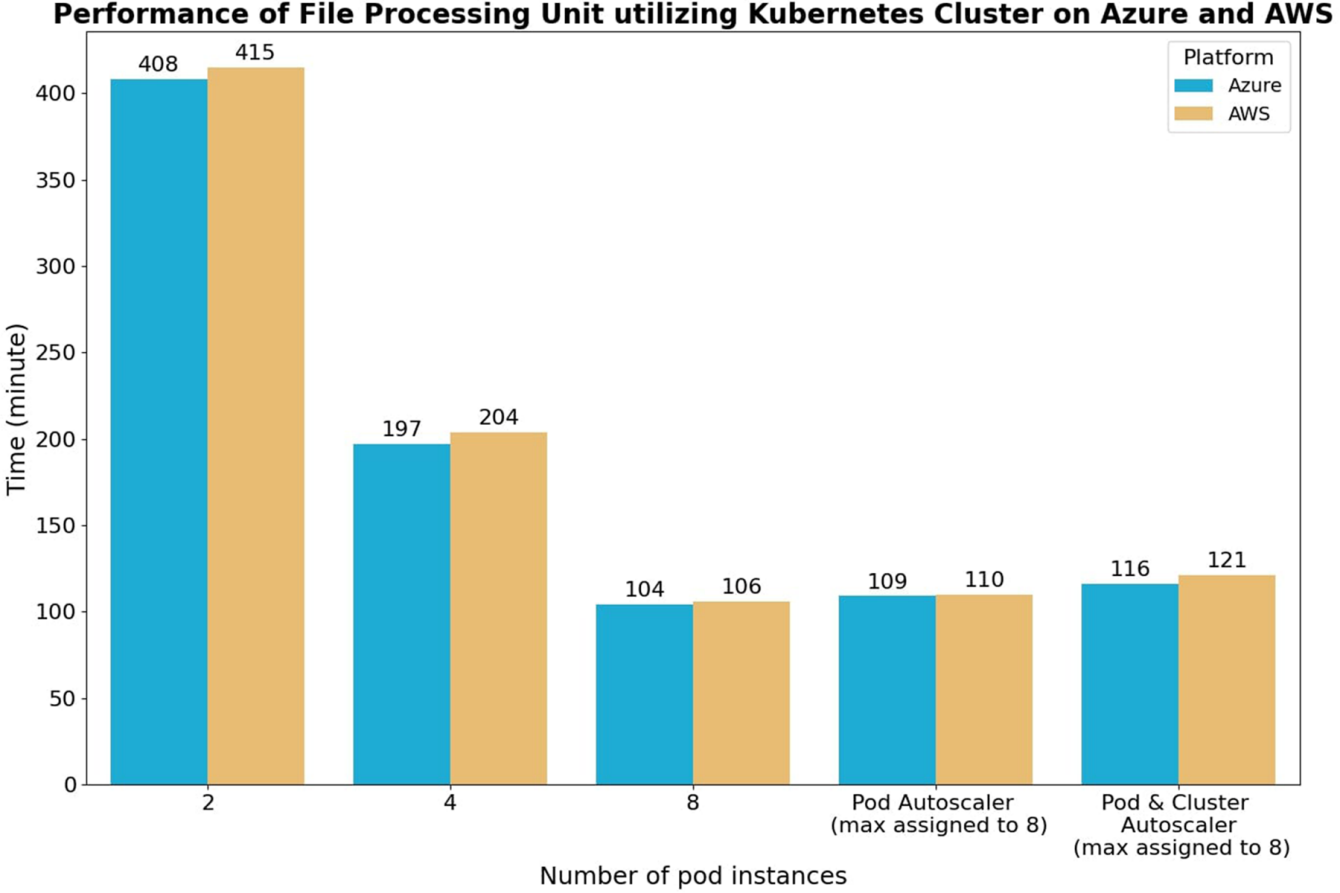
Performance of kubernetes cluster on AWS and azure platform using manual scaling, pod autoscaler, and pod + cluster autoscaler. The test processes 1,000 accelerometer raw data with a sampling frequency of 16 Hz and an average size of 1.5 MiB. The first three test cases are scaled by manually setting the number of running file processing instances with five nodes available. In the fourth test case, the horizontal pod autoscaler case starts with one pod instance and five nodes available. The last test case starts with one pod and one node that will be scaled up by both the horizontal pod and cluster autoscaler.

The test results, as depicted in Figure 8, demonstrate a linear increase in processing speed as the number of running instances doubles, indicating the effectiveness of parallel execution in the data processing unit pods. The horizontal pod autoscaler test case exhibited a delay of approximately 4–5 min compared to the manual eight-pod test case. This delay can be attributed to the scaling procedure of the horizontal pod autoscaler, which involves detecting resource constraints, allocating additional computing resources, and initializing the extra pods. In the horizontal pod and cluster autoscaler test case, a further delay of 3–4 min was observed compared to the horizontal pod autoscaler only test case. This delay arises from the scale-up procedure being performed across multiple VM nodes. Overall, these test cases highlight three important observations:

a) The management tasks of Kubernetes incur insignificant overhead time, as evidenced by the manual test case exhibiting results close to perfect scaling. This indicates that the Kubernetes system efficiently handles the scaling process.
b) The time required for both types of autoscalers to add more pods and nodes to the system is approximately 10–15 min. Although this delay may be negligible when the system is running for an extended duration, it can pose challenges during sudden bursts in processing demand. To address this, the queue messaging system acts as a buffer layer, cushioning the impact of client requests’ sudden spikes and preventing system overload. This buffer allows the autoscalers sufficient time to scale up the system to accommodate the increasing workload.
c) The VM computing performance between the Azure and AWS platform is similar to each other. The difference in performance is within the 5% margin of error.

The results of the data processing performance test highlight those applications managed by the Kubernetes system, with proper parallelization and adequate computing resources, exhibit a linear relationship between processing speed and the number of running pods. The small delay in scaling up computing resources is negligible under normal workload conditions. The automated scaling process enables the system to efficiently handle varying computing demands without wasting resources, ensuring optimal performance and scalability.

### 8.2 Database layer throughput performance test

The database layer throughput performance test aims to assess the scalability of the database in handling an increasing workload from concurrent clients. The test focuses on two key parameters: average latency of requests and the number of transactions per second, which are crucial metrics for measuring database throughput. To evaluate the throughput performance, we utilize the Pgbench tool provided by PostgreSQL 10 software (27), following the standard proposed by the Transaction Processing Performance Council (28). To isolate the impact of scaling up different factors, a specific test case is designed. Scaling up the database involves increasing both the number of CPU cores and the size of RAM. In this test, the Pgbench tool generates four tables to simulate the workload, as shown in Table 4.

**Table 4:** Pgbench parameters for the database throughput performance test.

Table name	Number of rows
pgbench_branches	1 × scale factor
pgbench_tellers	10 × scale factor
pgbench_accounts	100,000 × scale factor
pgbench_history	0

The data storage table is appropriately scaled to accommodate the variance in RAM size. Each scale factor is associated with a 13.5 MiB increase in table size. Consequently, the scale factor is determined such that the Pgbench test accesses data that matches the RAM usage of the respective test scenarios. For instance, to establish the scale factor for testing a scenario where the data accessed amounts to 10% of the Azure database's RAM, the scale factor is calculated as 10% of 10.2 GiB (the available RAM in the Azure setup) divided by 13.5 MiB, resulting in a scale factor of 75. The performance of the database service will be evaluated in two scenarios using the computed scale factor, as presented in Table 4. This evaluation will illustrate how system throughput is affected by RAM usage:

a) **Small**: The dataset size is set to 10% of the RAM capacity. Throughout the test, all accessed data is continuously stored in memory.
b) **Large**: The dataset size is set to 400% of the RAM capacity. Whenever the amount of data accessed during the test exceeds the RAM capacity, the operating system swaps the data from memory to disk.

The Pgbench query test is conducted on separate VMs located in the same region and deployed on the same cloud provider as the corresponding database. This setup ensures that any network distance-related variables are eliminated. Please refer to Table 5 for a detailed description.

**Table 5:** Configuration of the database instance on the azure and AWS platform used for the database throughput performance test.

Cloud platform	Azure	AWS
Database VM Instance name	General-Purpose Gen 5	db.m5.large
Cores	2	2
RAM	10.2 GiB	8 GiB
Scale factor (small dataset)	75	60
Scale factor (large dataset)	3,000	2,400
VM-test instance name	Standard DS4v2	m5d.2xlarge
VM-test cores	8	8
VM-test RAM	28 GiB	32 GiB

**Figure 9:**
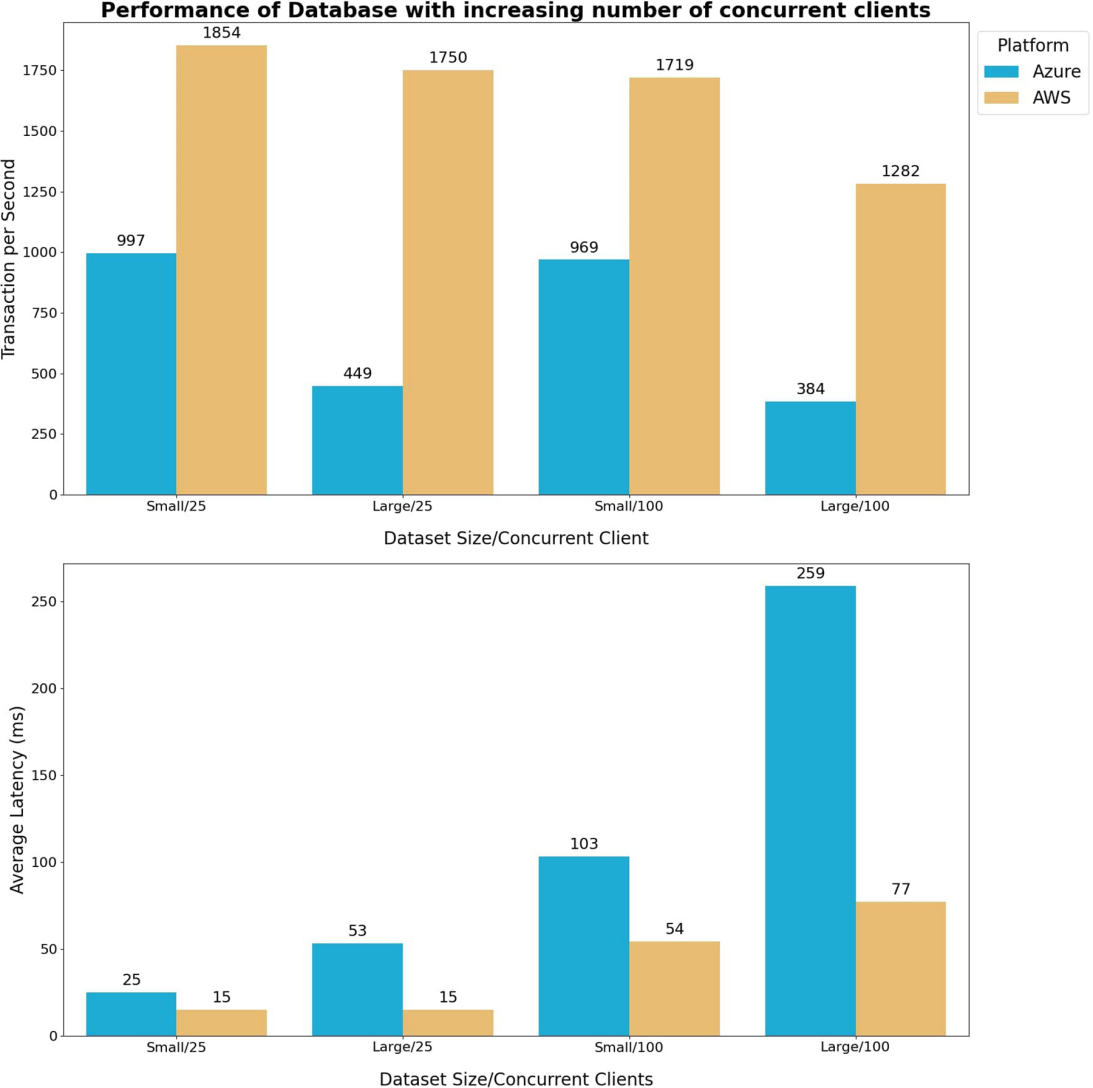
The database query performance test compares the performance of azure and AWS. The size of the test table in the database varies based on the test scenario and the available RAM capacity of the respective database. Two test scenarios are considered: small and large dataset. Small dataset scenario = 10% RAM capacity. Large dataset scenario = 400% RAM capacity. The query test is done using the Pgbench default test. Each query contains five commands: (1) update a random item from the pgbench_branches table, (2) select the item that was just updated from the pgbench_branches (3) update a random item from the pgbench_tellers table, (4) update 1 random item from the pgbench_accounts table, (5) insert a random item into the pgbench_history table. Each test case runs for an hour, and the results are averaged over three runs to mitigate variations. The objective of this test is to observe the impact of the number of concurrent clients and the data size in each transaction on the following metrics in the database: throughput, latency, and the number of transactions per second.

In Figure 9, the outcomes of the throughput performance test conducted at the database layer are illustrated. The results reveal a marginal reduction in transaction rates with an increase in the number of concurrent clients, while a substantial decline occurs when dealing with larger dataset sizes. This can be attributed to the faster access of data stored in memory, where smaller dataset minimizing the time that the database spent swapping data between disk storage and RAM. At the same time, the query latency increases with both the number of concurrent clients and the dataset size expands.

The performance test also shows the AWS platform outperforms the Azure platform in terms of throughput performance, given similar database configurations. This performance gap is primarily attributed to the AWS database showcasing significantly lower query latency compared to its Azure counterpart, even when both databases and the test VM simulating query requests are located within the same region (California - US West region).

## 9 General tips on healthcare cloud system design process

### 9.1 Emphasis on system modularity

Healthcare cloud systems face varying demands and must comply with specific regulations, so developers should prioritize modularity in system design. A modular approach enhances flexibility, making it easier to adapt to diverse requirements, such as:

• **Healthcare Integration Compliance:** Healthcare providers often use software from established vendors, making interoperability a challenge even with existing open-source standards like HL7 (29). By designing a system with modular interfaces, modifications can be limited to the interface layer, to ensure compatibility with different healthcare providers, as discussed in Section 4.
• **System Demand Changes:** Requirements and workloads can vary significantly over a project's lifecycle. For instance, a project may contain different phases, where phase one might focus on data collection, while phase two emphasizes on data analysis. The framework proposed in this paper allows for scaling the data storage and database units (Sections 5.1, 6) during data collection phase and then adjusting the data processing unit (Section 5) for analytics. If the data source transitions to a new healthcare provider, only the interface layer requires modification, while the rest of the system remains unaffected.
• **Software Updates:** Modular design enables individual components to be updated independently, allowing for rapid patching of critical vulnerabilities and reducing downtime.

### 9.2 Extensive use of fine-grained access authorization

Since social engineering is among the top causes of security breach (30), system designer should anticipate and mitigate the damage that is caused by bad actors having access to the system credential. Therefore, it is very important to limit each module to only being able to access what is needed to contain the scope of an unfortunate security breach. Some examples of fine-grained access control discussed in our framework are:

• **Sensor Management Unit**: Devices connected to the IoT hub are restricted to reading their configuration properties and uploading data to each device's own designated folder. They cannot read other devices’ data or modify their own.
• **Visualization Unit**: Users can only view their own data, while healthcare providers can access data for their assigned users. Administrative accounts have broader access but are advised to be used only in emergencies.
• **Data Processing Unit**: As this unit has access to all users’ raw data, it is recommended to configure it to operate within a firewall-isolated environment, with a defined IP address range. This setup restricts data access to registered IP addresses, ensuring that even in the event of credential compromise, unauthorized access is prevented. Additionally, database access is limited to requests originating from recognized IP addresses.

### 9.3 Regular backup and recovery

Given the rise of ransomware in recent years (31), having a robust backup and recovery plan is crucial. Cloud platforms provide configurations for regular backups in data storage (Sections 5.1.2, 6.2). To safeguard against worst-case scenarios, such as administrative account compromise, consider offline backups not connected to the internet. Providers like Azure and AWS offer hard drive shipping services, where data can be physically backed up onto hard drives and delivered to secure locations with ID verification. This approach serves as the final line of defense against system-wide ransomware attacks.

## 10 Conclusion

This paper addresses the challenges associated with collecting, processing, analyzing, and visualizing the vast amount of raw data generated by wearable sensor devices used for continuous patient monitoring. We recognize the critical importance of scalability in handling such large volumes of data and the need for robust security measures to protect patients’ health information (PHI) in accordance with the Health Insurance Portability and Accountability Act (HIPAA) guidelines.

To overcome these challenges, we propose a tailored Internet of Things (IoT) architecture specifically designed for the remote healthcare domain. Our approach leverages the power and capabilities of widely available commercial cloud platforms, namely Microsoft Azure and Amazon Web Services (AWS), while ensuring compliance with HIPAA regulations. By capitalizing on the scalability, load balancing, monitoring, and security functionalities offered by Azure and AWS, we aim to streamline the development process of creating HIPAA-compliant infrastructures for remote patient monitoring.

Furthermore, we conducted a comprehensive investigation into the data processing speed and database query latency of both Azure and AWS platforms. Through comparative analysis, we evaluated their performance at similar throughputs and computing powers when scaling up. This evaluation provides valuable insights into the capabilities and efficiency of these cloud platforms in handling the demands of healthcare data processing.

Overall, our proposed IoT architecture offers a practical and scalable solution for managing remote patient monitoring data while ensuring HIPAA compliance. By leveraging the capabilities of Azure and AWS, healthcare providers and developers can benefit from the extensive features and services offered by these cloud platforms, allowing for efficient, secure, and compliant remote healthcare systems. Future research can focus on exploring additional cloud platforms and evaluating their suitability for remote patient monitoring applications.

## Data Availability

The original contributions presented in the study are included in the article/Supplementary Material, further inquiries can be directed to the corresponding author.
